# Plant Defensins NaD1 and NaD2 Induce Different Stress Response Pathways in Fungi

**DOI:** 10.3390/ijms17091473

**Published:** 2016-09-03

**Authors:** Peter M. Dracatos, Jennifer Payne, Antonio Di Pietro, Marilyn A. Anderson, Kim M. Plummer

**Affiliations:** 1 Plant Breeding Institute Cobbitty, The University of Sydney, Private Bag 4011, Narellan, NSW 2567, Australia; 2 La Trobe Institute for Molecular Science, La Trobe University, Melbourne, VIC 3086, Australia; ja2payne@gmail.com (J.P.); m.anderson@latrobe.edu.au (M.A.A.); 3 Departamento de Genética, Campus de Excelencia Internacional Agroalimentario ceiA3, Universidad de Córdoba, Córdoba 14071, Spain; ge2dipia@uco.es; 4 Department of Animal, Plant and Soil Sciences, AgriBio, La Trobe University, Bundoora, VIC 3083, Australia; k.plummer@latrobe.edu.au

**Keywords:** antifungal defensins, NaD1 and NaD2, CWI signalling pathway, HOG signalling pathway

## Abstract

*Nicotiana alata* defensins 1 and 2 (NaD1 and NaD2) are plant defensins from the ornamental tobacco that have antifungal activity against a variety of fungal pathogens. Some plant defensins interact with fungal cell wall *O*-glycosylated proteins. Therefore, we investigated if this was the case for NaD1 and NaD2, by assessing the sensitivity of the three *Aspergillus nidulans* (*An*) *O*-mannosyltransferase (*pmt*) knockout (KO) mutants (*An*∆*pmtA*, *An*∆*pmtB*, and *An*∆*pmtC*). *An*∆*pmtA* was resistant to both defensins, while *An*∆*pmtC* was resistant to NaD2 only, suggesting NaD1 and NaD2 are unlikely to have a general interaction with *O*-linked side chains. Further evidence of this difference in the antifungal mechanism was provided by the dissimilarity of the NaD1 and NaD2 sensitivities of the *Fusarium oxysporum* f. sp. *lycopersici* (*Fol*) signalling knockout mutants from the cell wall integrity (CWI) and high osmolarity glycerol (HOG) mitogen-activated protein kinase (MAPK) pathways. HOG pathway mutants were sensitive to both NaD1 and NaD2, while CWI pathway mutants only displayed sensitivity to NaD2.

## 1. Background

Plant defensins are small, basic, cysteine-rich proteins that are often produced as a first line of defense against fungal attack [1,2,3]. Both NaD1 and NaD2 from the ornamental tobacco (*Nicotiana alata*) have antifungal activity, although NaD1 is more active on most fungal species [2,4]. More is known about the mechanism of action of NaD1, compared to NaD2. The antifungal activity of NaD1 involves a multi-step mode of action; whereby NaD1 interacts specifically with the fungal cell wall and ultimately enters the cytoplasm [5], resulting in the disruption of the plasma membrane and the production of reactive oxygen species [6]. NaD2 has also been shown to permeabilise the plasma membrane of *Puccinia* spp. [4].

NaD1 and NaD2 share only 40% sequence identity and 51% sequence similarity and have different mechanisms of antifungal activity to each other [4,7]. One obvious difference is that the two defensins interact with different phospholipids in bilayers; NaD1 with phosphatidylinositol 4,5 bisphosphate (PI(4,5)P_2_) [8,9] and NaD2 with phosphatidic acid (PA) [7]. To reach the fungal plasma membrane, NaD1 and NaD2 must first pass through the wall. However, it is not known whether both NaD1 and NaD2 interact with the same components of the cell wall or induce the same cellular response pathways in fungi.

The glycoprotein layer of the fungal cell wall has been implicated in the antifungal activity of NaD1, because proteinase K treated fungi are resistant to NaD1 [5]. These cell wall glycoproteins are often heavily glycosylated with both *N*- and *O*-linked side chains. A genetic screen of *Candida albicans* revealed that mutations in the α-1,6-mannosyltransferase complex, which is involved in the *O*-mannosylation of cell wall proteins, resulted in resistance to dermaseptin S3 and the plant defensin *Pharbitis* *nil* antimicrobial peptide 1 (Pn-AMP1) [10,11]. We have used a similar approach to examine whether *O*-linked side chains are involved in the antifungal activity of NaD1 and NaD2, by assessing the sensitivity of *Aspergillus nidulans O*-mannosyltransferase knockout strains. *O*-mannosyltransferase catalyze the initial transfer of mannose onto the serine or threonine to start the *O*-linked side chain of the protein. *A. nidulans* is ideal for this work as it possesses a single gene for each of the *O*-mannosyltransferase sub-families (*pmtA, pmtB* and *pmtC*) and mutants are viable [12]. These *pmt* mutants have different phenotypes suggesting that the enzymes have distinct protein substrates [13,14].

Cell wall glycoproteins have roles in a variety of cellular functions including signaling, protection, and coping with cell stress [15]. The fungal response to antifungal peptides is often sensed and initiated through these glycoproteins, leading to the activation of the mitogen-activated cell wall integrity (CWI) pathway or the high osmolarity glycerol (HOG) pathway [16,17,18,19]. Studying the pathways activated by antifungal peptides gives an insight into their mechanism of action. For example, a recent study in *C. albicans* revealed that the HOG signalling pathway plays a role in the cellular tolerance and response to NaD1 as exposure to NaD1 leads to oxidative stress through the production of reactive oxygen species [6]. In contrast the plant defensins *Raphanus sativus* antifungal peptide 2 (RsAFP2), and *Medicago sativa* defensin 1 (MsDef1), activate the CWI pathway in *C. albicans* and *Fusarium graminearum* respectively [20,21], possibly due to their interaction with the cell wall sphingolipid, glucosylceramide [20]. However, it is not known what pathways are activated in response to NaD2. Therefore, the activation of cell stress responses to NaD1 and NaD2 was also investigated by testing the sensitivity of *F. oxysporum* f. sp. *lycopersicum* (*Fol*) KO mutants of the CWI and HOG pathways.

## 2. Results

### 2.1. Impact of Plant Defensins, NaD1 and NaD2, on the Growth of Aspergillus nidulans (A. nidulans) O-Mannosyltransferase Knockout (KO) Mutants 

**Figure 1:**
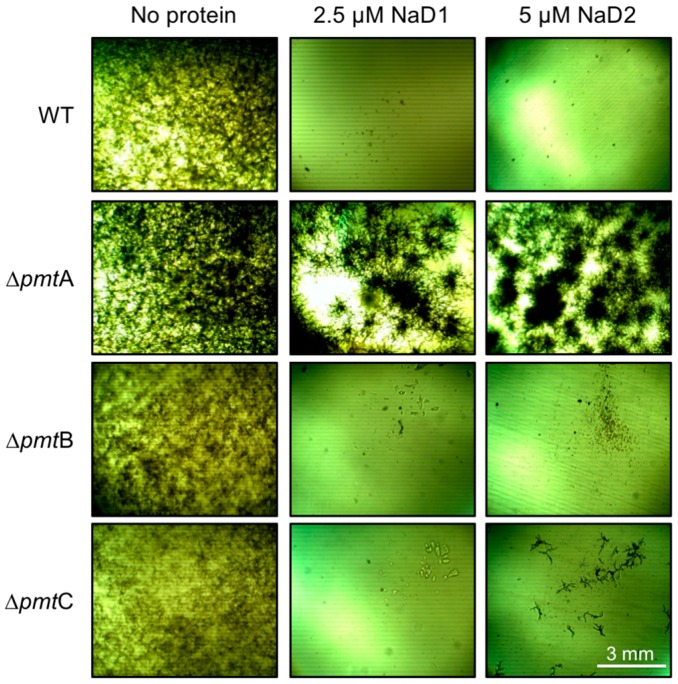
Growth of the *A. nidulans* (*An*) *O*-mannosyltransferase (*pmt* *A*, *B* or *C*) knockout mutants (*An*∆*pmtA, An*∆*pmtB or An*∆*pmtC*) in the presence/absence of plant defensins NaD1 (2.5 µM) and NaD2 (5 µM). Images are representative of three experiments.

Disruption of the main *O*-mannosyltransferase in *A. nidulans*, *An*∆*pmtA*, led to enhanced resistance to both NaD1 and NaD2, compared to the WT, *An* strain A850 (Figure 1). A limited amount of growth of the *O*-mannosyltransferase C mutant, *An*∆*pmtC,* was observed in the presence of NaD2, but not with NaD1. In all experiments, the sensitivity of the *O-*mannosyltransferase B KO mutant, *An*∆*pmtB,* closely resembled that of the WT*.* All knockout mutants grew as per the wild type strain in the absence of defensins (Figure 1).

### 2.2. Defensin Growth Inhibition of the Fusarium oxyspoprum f. sp. lycopersici Mitogen-Activated Protein Kinase (MAPK) Pathway KO Mutants

**Figure 2:**
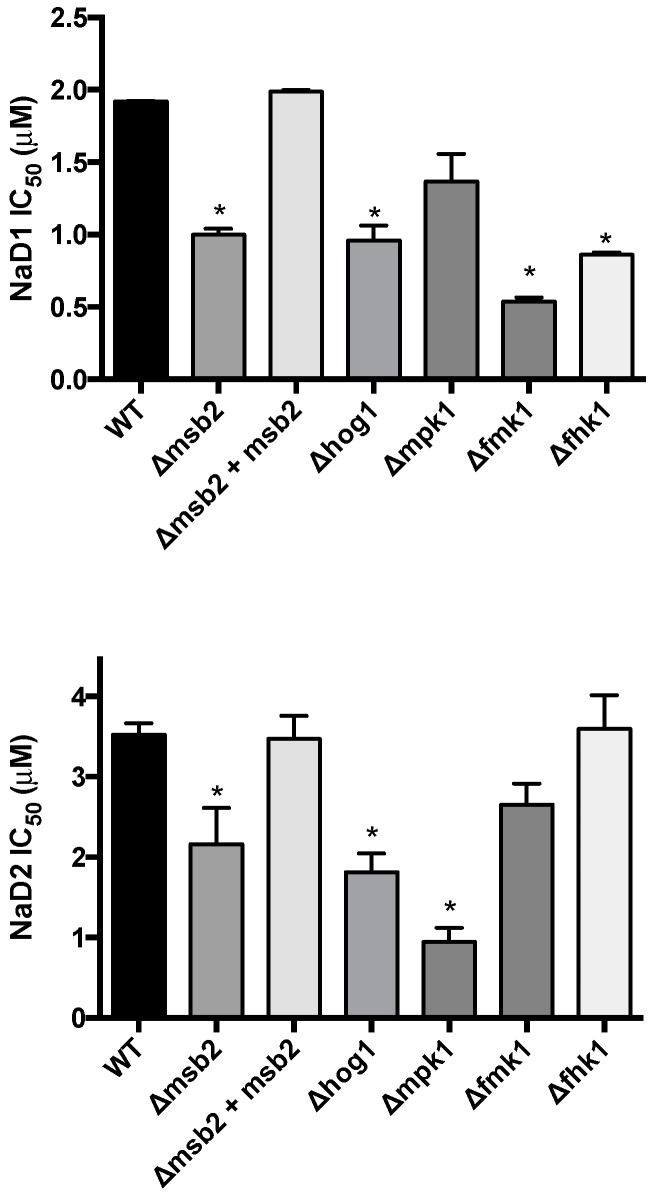
Concentrations of the plant defensins, NaD1 and NaD2, required to inhibit 50% growth (IC_50_) of *Fusarium oxysporum* f. sp. *lycopersici* knock out mutants of the mitogen-activated protein kinase (MAPK) pathways. Error bars are SEM, NaD1 *n* = 3, NaD2 *n* = 6. * indicates data that is significantly different (*p* < 0.05) to WT.

The sensitivity of *F. oxysporum* f. sp. *lycopersici* (*Fol*) knockout mutants of cellular response pathway genes were assessed to determine the potential involvement of mitogen-activated protein kinase (MAPK) cascades in enhancing tolerance to NaD1 and NaD2. Mutants lacking components of the HOG pathway, specifically the *Fusarium* histidine kinase 1 (Fhk1) and the MAPK Hog1, were both hypersensitive to NaD1, with a 2-fold decrease in IC_50_ compared to WT (Figure 2). In addition, the KO strains in the *Fusarium* MAPK 1 (*Fol*Δ*fmk1*) and the transmembrane mucin Msb2 (*Fol*Δ*msb2*) were also significantly hypersensitive to NaD1. Similarly, the *hog1* and *msb2* KO mutants were more sensitive to NaD2. However, the mutant (*Fol*Δ*mpk1*) lacking the stress response MAPK, Mpk1, exhibited a 4-fold decrease in IC_50_ compared to WT nd hence was more sensitive to NaD2 than NaD1 and conversely the *Fol*Δ*fmk1* and *Fol*Δ*fhk1* mutants were more sensitive to NaD1 than WT and not sensitive to NaD2. The complemented strain, *Fol*Δ*msb2* + *Msb2*, had a similar IC_50_ to that of the WT for both defensins.

## 3. Discussion

The plant defensins NaD1 and NaD2 have antifungal activity against a wide range of agronomically and medically important microbes, including *Fusarium* and *Aspergillus* species [4,22]. Understanding the mechanism of action of these defensins will therefore help to develop these antifungal peptides for agricultural and pharmaceutical use. Combining molecules with multiple mechanisms of action into one treatment has been suggested as a way to reduce the occurrence of resistance. NaD1 and NaD2 differ markedly in their amino acid sequence, especially in their γ-core [4]. The presence of different sequences in this area gives rise to different lipid specificity and antifungal mechanisms [23,24,25,26]. We therefore investigated the *N. alata* defensins to examine their differences in activity, to determine if the ornamental tobacco produces defensins with multiple mechanisms of action. This study was aimed at testing the hypothesis that NaD1 and NaD2 are likely to interact with distinct components of the fungal cell wall, and induce different cellular stress response pathways.

We tested the sensitivity of *A. nidulans* *O*-mannosyltransferases (*pmtA*, *pmtB* and *pmtC*) KO mutants in response to NaD1 and NaD2 to determine if *O*-linked side chains were involved in their antifungal activity. Assessing the sensitivity of the *A. nidulans* protein *O*-mannosyltransferase (PMT) mutants to both defensins revealed that only the deletion of the *pmtA* gene resulted in a mutant (*An*∆*pmtA*) with a resistant phenotype to both NaD1 and NaD2, compared to WT. No resistance to NaD1 and NaD2 was observed with the *An*∆*pmtB* mutant. Both defensins are therefore unlikely to have a general interaction with cell wall *O*-linked sided chains. This is in contrast to observations with the antifungal peptide dermaseptin S3 and the plant defensin Pn-AMP1, where *O*-linked side chains on glycoproteins have been implicated in their mechanism of action from mutant screening [10,11]. The mutant *An*∆*pmtC* was mildly resistant to NaD2 only, suggesting that the glycoprotein targets for NaD1 and NaD2 may be different. The selective resistance of just *An*∆*pmtA* to both defensins may be explained by examining the proteins that receive their *O*-linked side chains from PmtA. The *O*-mannosyltransferases in *A. nidulans* are believed to have overlapping and distinct specificities for protein substrates due to the distinct phenotypes of the mutants and their different sensitivities to antifungal agents [12,13]. The phenotype of *An*∆*pmtB* mutant suggests involvement in polarity maintenance, while the *An*∆*pmtC* phenotype suggests involvement in cell wall integrity [13]. The mannosyltransferases responsible for glycosylation of individual proteins are only known for a couple of proteins. For example, MsbA (an orthologue of Msb2 from *C.* *albicans* and *Fol*) is differentially glycosylated by PmtA and PmtB [27]. Msb2 is an osmosensor in the HOG pathway, and the HOG pathway has been implicated in the fungal response to NaD1, as deletion of the *hog1* gene generates sensitivity to NaD1 in *C.* *albicans* [6]. Another protein mannosylated by PmtA is WscA (an orthologue of Wsc1 in *Saccharomyces* *cerevisiae*) [13], which has a role in cell wall integrity under changes in osmotic and pH conditions [28]. The observed resistance phenotype of *An*∆*pmtA* to NaD1 and NaD2 may be due to the lack of mannosylation of the membrane mucin MsbA, and/or WscA, which leads to the defect in the signalling of the HOG and CWI pathways, rather than a direct interaction of the defensins with these receptors. However, further experiments are required to confirm this hypothesis.

The involvement of the HOG pathway in the activity of both NaD1 and NaD2 was supported as deletion of either *Msb2* or *Hog1* led to enhanced sensitivity to both defensins. Loss of the HOG pathway components would create a defect in the downstream signalling, resulting in a fungus that is more sensitive to defensin attack. This suggests that the HOG pathway is involved in tolerance of *Fol* to these two defensins. Therefore, the HOG pathway is not just activated in response to NaD1 in the human pathogen *C. albicans* [6], but also in the agronomically important pathogen, *Fol*. Modulation of the HOG pathway in combination with either NaD1 or NaD2 could be a promising strategy for increasing the activity of these defensins against pathogenic fungi. This has been suggested previously as a combinational strategy against human pathogens for the human antimicrobial peptides; histatin 5 and β-defensins 2 and 3, which also activate the HOG pathway [29,30]. Here we show that this could be extended to strategies to combat plant pathogens.

MAPK pathways, such as CWI pathway or the HOG pathway are responsible for the quick transduction of signals in response to environmental stresses, such as those caused by the antifungal defensins. The pathway activated depends upon the stimulus, and therefore can give insights into the mechanism of action of an antifungal peptide. For example, the fungal cell responds to some plant defensins by activating the CWI pathway, and these defensins have interactions with the cell wall as part of their activity. These include Pn-AMP1 which interacts with *O*-linked side chains resulting in activation of CWI pathway [10]; and MsDef1 and RsAFP2, which interact with glucosylceramide in the cell wall, causing CWI pathway activation [20,21]. In this present study, we observed that deletion of the CWI MAPK *Mpk1* lead to increased sensitivity to NaD2, but not to NaD1. It would therefore be interesting to investigate the direct interaction of NaD2 with the cell wall beyond the *O*-linked side chains to determine why NaD2 is activating the CWI pathway. Further difference in cell response pathways was also observed between NaD1 and NaD2 when the deletion of the histidine kinase Fhk1 and the pathogenicity MAPK Fmk1 lead to sensitivity to only NaD1. Therefore, the cell stress pathways activated by NaD1 and NaD2 in *Fol* appear to be different, providing further evidence that the two defensins have distinct mechanisms of action.

Taken together, sequence variations between NaD1 and NaD2 are likely to explain the differences in fungal mutant sensitivities observed. The HOG pathway appears to be involved in the stress response to both NaD1 and NaD2, however it remains to be determined if NaD2, like NaD1, activates this pathway through reactive oxygen species or, instead, through osmotic stress as occurs with the activity of histatin 5. To study differences in the mode of action of defensins in more detail, we are attempting to identify targets by screening yeast deletion libraries to identify genes that confer resistance or sensitivity to different defensins [31]. We are also determining whether fungal pathogens respond differently to different defensins by examining changes in the transcriptome of various fungal pathogens after exposure to sub-lethal amounts of defensins. In summary, NaD1 and NaD2 have evolved as part of the arsenal of innate immunity molecules that protect the ornamental tobacco against fungal disease. Their overlapping, as well as distinct modes of action are likely to provide activity against a broader range of fungal pathogens and make it more difficult for pathogens to become resistant to these defensins.

## 4. Methods

### 4.1. Fungal Strains and Media

The *A. nidulans* (*An*) and *F. oxysporum* f. sp. *lycopersici* (*Fol*) knockout (KO) mutants used in this study are listed in Table 1. Complete Medium (CM) was prepared essentially as described by Kriangkripipat and Momany [32]. In all cases the *A. nidulans* isolates were grown on minimal media that contained arginine; arginine and tryptophan; or arginine, tryptophan and methionine (depending on the specific mutant); and the pH was increased to 6.5 by addition of 1 M NaOH. For sporulation, *A. nidulans* strains were grown at 26 °C on CM media containing 1.5% agar and 1 M sorbitol as an osmotic stabilizer.

### 4.2. Protein Source

NaD1 and NaD2 were extracted from the flowers of ornamental tobacco as described in van der Weerden, Lay and Anderson [22].

### 4.3. Fungal Growth Assays

Fungal growth assays were performed in microtitre plates and the concentrations of defensins required to inhibit fungal growth by 50% (IC_50_) were calculated as described in van der Weerden, Lay and Anderson [22]. IC_50_ values were plotted using Graphpad Prism v6 (GraphPad Software, La Jolla, CA, USA) and *t*-tests with Welch′s corrections were performed to determine significance of the growth data of each mutant compared to WT. Calculation of the IC_50_ was not possible for the *A. nidulans* mutants due to uneven growth. Instead the growth of the *A. nidulans* mutants in the presence of plant defensins was assessed visually by capturing images using an Olympic microscope at 10× magnification. The growth assays were conducted in the dark at 25 °C for 48 h.

## Figures and Tables

**Table 1:** List of fungal strains used in this study.

Fungal Species	Genotype/Phenotype	Protein	Source or Reference
*Aspergillus nidulans*	Wild Type A850*-arg::trpC_B methG*	–	Kriangkripipat and Momany [32]
	ATK08-*pyrG89 argB2::trpC B pyroA4 ΔpmtA::AfpyrG*	*O*-mannosyltransferase subfamily pmtA	Kriangkripipat and Momany [32]
	ATK16-*pyrG89 ΔpmtB::AfpyrG argB2 pyroA4*	*O*-mannosyltransferase subfamily pmtB	Kriangkripipat and Momany [32]
	ATK38-*pyrG89 wA3 argB2 pyro A4 ΔpmtC::AfpyrG*	*O*-mannosyltransferase subfamily pmtC	Kriangkripipat and Momany [32]
*F. oxysporum* f. sp. *lycopersici*	race 2 wild-type strain 4287 (FGSC 9935)	–	Di Pietro, et al. [33]
	*Δhog1*	High osmolarity glycerol MAPK	Luque et al. [34]
	*Δmsb2*	Glycosylated receptor of the HOG pathway	Perez-Nadales and Di Pietro [15]
	*Δmsb2 + Msb2*	Complemented Msb2	Perez-Nadales and Di Pietro [15]
	*Δmpk1*	Cell wall integrity MAPK	Turra, et al. [35]
	*Δfmk1*	Pathogenicity MAPK	Di Pietro, Garcia-MacEira, Meglecz and Roncero [33]
	*Δfhk1*	Histidine kinase	Rispail and Di Pietro [36]
